# Diagnostic performance of *Strongyloides*-specific IgG4 detection in urine for diagnosis of human strongyloidiasis

**DOI:** 10.1186/s13071-023-05935-6

**Published:** 2023-08-28

**Authors:** Phattharaphon Wongphutorn, Chanika Worasith, Kulthida Y. Kopolrat, Chutima Homwong, Jiraporn Sithithaworn, Chatanun Eamudomkarn, Anchalee Techasen, Patcharaporn Tippayawat, Opal Pitaksakurat, Nuttanan Hongsrichan, Thomas Crellen, Paiboon Sithithaworn

**Affiliations:** 1 Biomedical Science Program, Graduate School, Khon Kaen University, Khon Kaen, Thailand; 2 Department of Adult Nursing, Faculty of Nursing, Khon Kaen University, Khon Kaen, Thailand; 3 Faculty of Public Health, Kasetsart University Chalermphrakiat Sakon Nakhon Province Campus, Sakon Nakhon, Thailand; 4 Cholangiocarcinoma Research Institute, Khon Kaen University, Khon Kaen, Thailand; 5 Faculty of Associated Medical Sciences, Khon Kaen University, Khon Kaen, Thailand; 6 Department of Parasitology, Faculty of Medicine, Khon Kaen University, Khon Kaen, Thailand; 7 School of Biodiversity One Health and Veterinary Medicine, University of Glasgow, Graham Kerr Building, Glasgow, UK; 8 Wellcome Centre for Integrative Parasitology, University of Glasgow, Sir Graeme Davies Building, Glasgow, UK; 9 Big Data Institute, Li Ka Shing Centre for Health Information and Discovery, University of Oxford, Oxford, UK

**Keywords:** *Strongyloides stercoralis*, Enzyme-linked immunosorbent assay (ELISA), Immunoglobulin G4

## Abstract

**Background:**

Detection of parasite-specific IgG in urine is a sensitive method for diagnosis of strongyloidiasis and gives similar accuracy to serum IgG. However, there are no data concerning detection of IgG subclass in urine. To further explore the utility of diagnosis from urine samples, we evaluated the diagnostic performance of IgG4 in urine compared with parasitological and other immunological methods.

**Methods:**

The urine and sera included proven strongyloidiasis (group 1, *n* = 93), other parasitic infections (group 2, *n* = 40) and parasite negatives (group 3, *n* = 93). The performance of *Strongyloides*-specific IgG4 in urine for diagnosis of strongyloidiasis using fecal examinations as the reference standard was assessed.

**Results:**

With fecal examination as a gold standard, *Strongyloides*-specific IgG4 in urine had 91.4% sensitivity and 93.2% specificity while serum IgG4 had 93.6% sensitivity and 91.0% specificity. IgG4 in both urine and serum had almost perfect diagnostic agreements with fecal examination (Cohen's kappa coefficient was > 0.8). Cross-reactivity to *Opisthorchis viverrini* and *Taenia* spp. of IgG4 in urine were 7.5% and 12.5% in serum. Concurrent analyses of total IgG in urine and serum showed that the sensitivities (97.9–100%) and specificities (88.7–91.0%) were similar (*P* > 0.05). The sensitivity for parasitological examination by the formalin-ethyl acetate concentration technique (FECT) was 49.5% and that for agar plate culture technique (APC) it was 92.6%.

**Conclusion:**

Our findings showed that specific IgG4 detection in urine yielded similar diagnostic performance to the same biomarkers in serum. This suggests that accurate diagnosis of strongyloidiasis can be performed using urine samples and IgG4 is a valid choice of diagnostic marker. Further assessment is required to assess the utility of urine IgG4 for measuring the response treatment in strongyloidiasis.

**Graphical Abstract:**

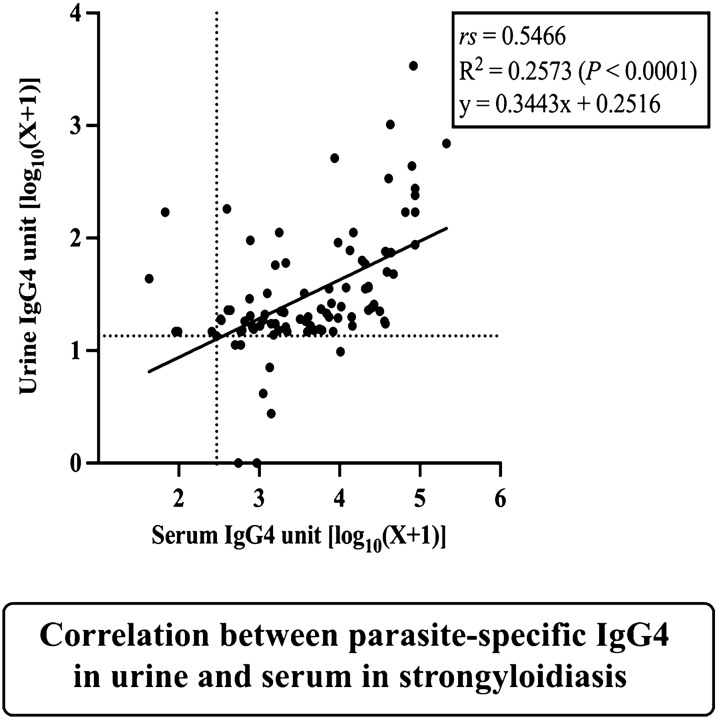

**Supplementary Information:**

The online version contains supplementary material available at 10.1186/s13071-023-05935-6.

## Background

Strongyloidiasis is a neglected tropical disease (NTD) caused by infection with the nematode *Strongyloides stercoralis*. Transmission of the parasite occurs globally in tropical and subtropical regions [1, 2]. In Southeast Asia, *S. stercoralis* infections in humans have been reported in Thailand, Lao PDR, Vietnam and Cambodia [1–4]. The potential for autoinfection exists when developing filariform larvae of *S. stercoralis* reinfect the same host, leading to lifelong infection. Strongyloidiasis is normally asymptomatic but occasionally has manifestations including gastrointestinal and dermatological symptoms; in cases where host immunity is compromised, hyperinfection and fatal disseminated strongyloidiasis can occur [5–7].

Sensitive and specific diagnosis of *S. stercoralis* infection is required for initiation of treatment to prevent disease complication as well as to improve the surveillance and control of strongyloidiasis. Fecal examinations, such as the agar plate culture (APC) and Baermann’s technique, are considered “gold-standard” diagnostic methods for *S. stercoralis* infection. However, both have low sensitivity due to the frequently low density of larvae and high daily fluctuation of larval output in feces. Repeated fecal examination is recommended to improve sensitivity, but it can be logistically difficult for patients and laboratory staff [8, 9]. To alleviate the low sensitivity, several alternative diagnostic methods have been developed, mostly using immunological methods such as antigen or antibody detection in serum, saliva and urine [10–13]. Molecular methods have also been used to detect *S. stercoralis* DNA with varying sensitivity in fecal (75–100%) and urine specimens (77.1%) and specificity (67.8% in urine and 80–90% in feces) [14, 15]. Costly reagents and specialized facilities are required for molecular methods. Therefore, in countries where resources are limited and strongyloidiasis is prevalent, it is essential to develop more convenient and inexpensive methods [14–16].

Collection of urine samples is non-invasive and more convenient for patients than collection of blood or fecal samples. Detection of parasite-specific IgG antibodies in urine was recently established as a method for the diagnosis of strongyloidiasis and yielded diagnostic accuracy similar to serum assays, both of which are more sensitive than fecal examination [8, 11]. Previous studies reported that several parasitic infections induced high IgG4 responses which were associated with asymptomatic and chronic infections including filariasis, strongyloidiasis and schistosomiasis [17–21]. Detection of specific IgG4 has been used for diagnosis of strongyloidiasis using different platforms including the lateral-flow assay and ELISA platform [18, 20, 22–25]. Parasite-specific IgG4 is known to be highly specific for the diagnosis of strongyloidiasis, especially in chronically infected individuals [13]. Moreover, IgG4 has been used to assess the efficacy of drug treatment: a sustained level of specific IgG4 after albendazole treatment indicated treatment failure for strongyloidiasis [26, 27]. However, detection of *Strongyloides*-specific IgG4 in urine for diagnosis of strongyloidiasis has not been reported. We hypothesize that measurements of IgG4 in urine has similar qualitative and quantitative diagnosis to those in serum in strongyloidiasis.

This study aims to develop an indirect ELISA for detection of specific IgG4 in urine samples and to evaluate the diagnostic performance of this approach in strongyloidiasis. Diagnostic agreement and quantitative correlations between specific IgG4 in serum and urine will be analyzed. In addition, diagnostic performance of specific total IgG in serum and urine detection will be evaluated with reference to the fecal examination by the agar plate culture technique (APC) and the formalin-ethyl acetate concentration technique (FECT) as gold standard methods. The results in this study highlighted that detection of specific IgG4 in urine had high diagnostic performance similar to that of serum IgG4 and had higher specificity than total IgG in serum and urine samples, indicating its potential as an immunological marker for diagnosis of strongyloidiasis.

## Methods

### Study participants and specimen collection

This study was a cross-sectional study for screening of strongyloidiasis using parasitological and immunological methods under the long-term cohort study of opisthorchiasis. The eligibility criteria for recruitment and study inclusion were (i) participants were native residents in Thung Chomphu subdistrict, Phuwiang district, in Khon Kaen province, northeast of Thailand; (ii) participants agreed to donate clinical samples including feces, blood and urine for index and standard reference tests; (iii) participants were currently healthy and had no clinical signs or symptoms of liver or kidney diseases. As it is more acceptable to collect urine samples from the study population compared with blood/serum, we used a non-inferiority trial framework for sample size calculations where the expected sensitivity was 95% for serum IgG and 90% for urine IgG. Given an acceptable non-inferiority margin of 5% between serum and urine diagnostics, we calculated that a sample size of 55 positive individuals is sufficient to determine non-inferiority between the two diagnostics using a one-sided significance threshold of 5%. If the non-inferiority margin is widened to 10%, then the required number of positive cases drops to 25 individuals. Given an a prior expected prevalence of *S. stercoralis* in the community as 30%, this gave a required sample size at recruitment of 183 participants.

**Fig. 1:**
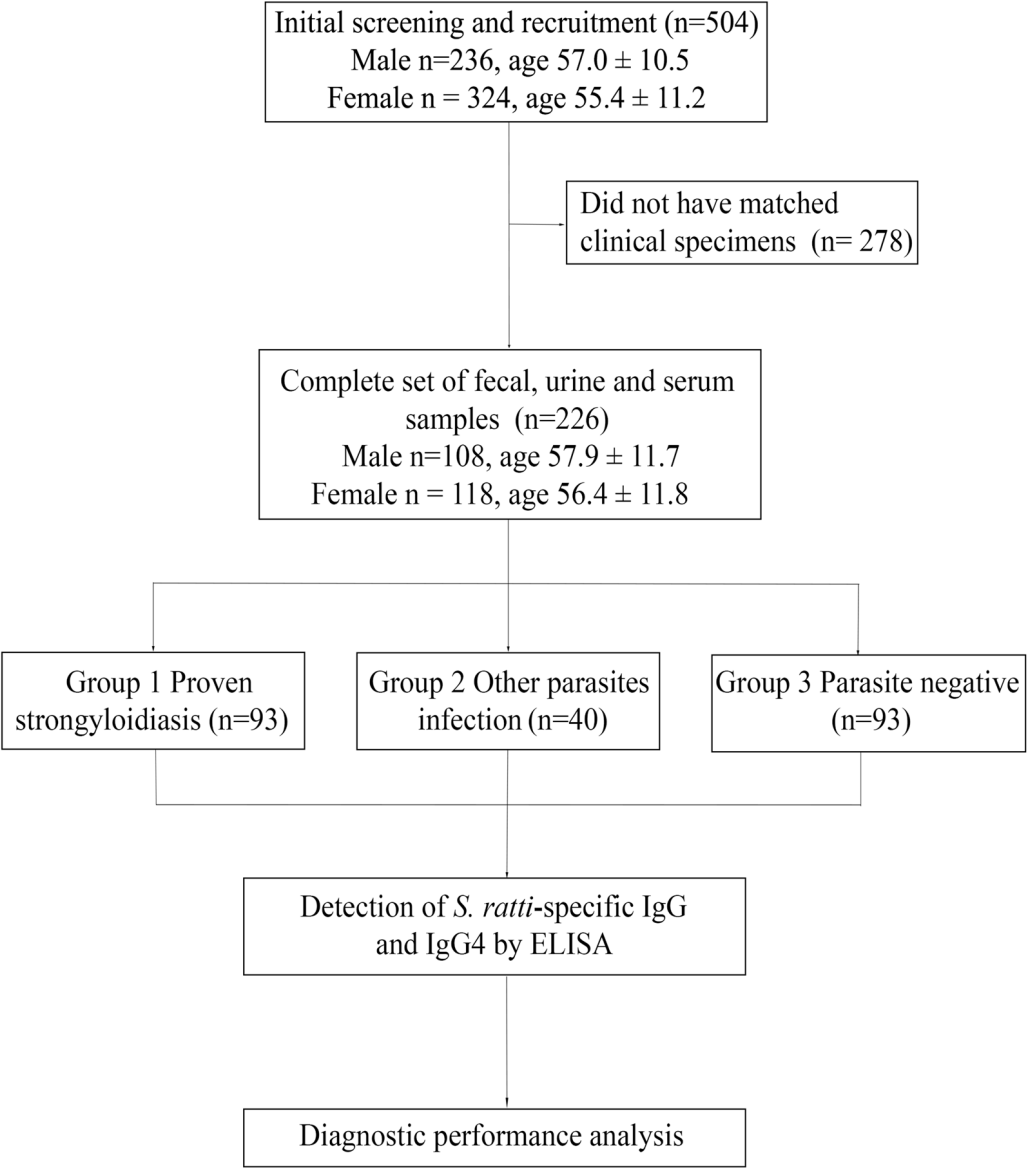
Flow diagram of the study approach showed the number of participants divided into three groups: Group 1, proven strongyloidiasis with *Strongyloides stercoralis* larvae in feces; Group 2, other parasite infections; Group 3 parasite negatives

A total of 504 participants fulfilled the inclusion criterion, and 226 provided complete clinical samples (Fig. 1). There was no statistical difference in demographic data (age and sex) between enrolled participants and the number of individuals who submitted complete sets of specimens. In groups of *S. stercoralis* larvae negative, *Strongyloides*-specific IgG in serum was confirmed by NIE-based ELISA and Ss-based ELISA.

Recruited participants were requested to provide clinical samples, i.e. feces, urine and serum. Ten grams of fecal sample from each participant was collected in a clean wide-mouth container. Each fecal sample was separated into aliquots for APC and the formalin-ethyl acetate concentration technique (FECT). For the urine sample, 10 ml of first morning urine was obtained from each participant and was preserved in a test tube containing NaN_3_ (0.1%). Each urine sample was transferred into a microwell plate and kept at 4 °C until required. Five milliliters of blood from each participant was collected in 10-ml serum separating tubes (clot activator tube) (BD, UK). Aliquots of 500 µl were transferred to new 1.5-ml microcentrifuge tubes for determination of the level of total IgG and of IgG4 by ELISA.

### Fecal examination

The two fecal examination methods used to assess parasitic infections were FECT and APC. Two grams of fecal sample was processed for FECT [28]. In brief, formalin-fixed samples were filtered through gauze, and ethyl acetate was used as an extractor of fat from feces. After centrifugation at 500 × g for 5 min, the supernatant was removed, and the sediment was suspended in 1 ml 10% formalin. Three drops of the final fecal suspension were sampled and examined for parasites. The number of larvae per gram (LPG) of feces was calculated and presented based on 2 g feces. The APC method has long been considered a reference standard for detection of *S. stercoralis* [29]. Approximately 4 g of feces from each participant was placed on a 1.5% nutrient agar plate in a plastic Petri dish and incubated at 25 °C for 2–4 days. Plates were examined under a stereo microscope for the presence of worms. Then, the plate and the underside of each lid were washed with 10% formalin; the washings were transferred into a centrifuge tube and examined with a microscope to distinguish between *S. stercoralis* stages and hookworms. The laboratory staff who undertook the procedure were blinded for sample IDs and laboratory data of the participants.

The total number of fecal samples analyzed by FECT were 226, with 174 samples adequate for APC analysis. The samples from 52 participants were omitted from APC analysis because of inadequate quantity of the sample. The final number of fecal-positive samples used as proven strongyloidiasis was a combination of FECT and/or APC (*n* = 93).

### Measurements of specific IgG in urine and serum

The indexed tests were unit-based ELISA protocol for IgG; *Strongyloides ratti* as antigen, described previously, was used with slight modifications [11, 12]. The 96-well plates were coated with 2.5 ug/ml *S. ratti* antigen, respectively, at 4 °C overnight. The plates were washed twice using phosphate-buffered saline (PBS) containing 0.05% Tween-20 (pH 7.2) before blocking with 3% skimmed milk in PBS containing 0.5% Tween-20 at ambient temperature for 2 h. One hundred microliters of undiluted urine or diluted serum samples (diluted at 1:8000) was added and incubated at 37 °C for 1 h. After triplicate washing of the plates, 100 µl of horseradish peroxidase conjugate goat antihuman IgG (Abcam, USA) was added and incubated at 37 °C for 1 h. After being washed three times, the OPD substrate (Sigma-Aldrich, USA) in citrate–phosphate buffer pH 5.0 (100 µl/well) was added and the plates incubated at room temperature for 1 h in a humidified dark container. Finally, the reaction was stopped by adding 4 N sulfuric acid (50 µl/well), and optical density (OD) was measured at 492 nm using an ELISA reader (TECAN Sunrise, Austria). The OD was transformed to arbitrary antibody units (per ml urine or serum) based on a standard curve generated from serial dilutions of pooled positive serum samples [11, 12].

### Measurements of IgG4 in urine and serum

The optimal conditions for the IgG4 indexed tests in urine and serum were pre-determined in the optimization study. Crude *S. ratti* antigen (10 µg/ml) was used to coat wells in 96-well microtiter plates at 4 °C overnight. The plates were washed twice (30 s each time) with PBS pH 7.2 containing 0.05% Tween-20 before blocking with 3% skimmed milk in PBS containing 0.5% Tween-20 at ambient temperature for 2 h. The urine (100 µl undiluted) or diluted serum samples (100 µl of 1:40 dilution) were added to wells and incubated at 37 °C for 1 h. After triplicate plate washing, 100 µl of diluted horseradish peroxidase conjugate goat antihuman IgG4 (Invitrogen, USA) at 1:1000 dilution was added and incubated at 37 °C for 1 h. After three washes, 100 µl/well of the TMB (3,3',5,5'-tetramethylbenzidine) substrate was added and incubated at ambient temperature for 30 min in humidified dark containers. Finally, the reaction was stopped by adding 4 N sulfuric acid (50 µl/well), and OD values were measured at 450 nm using an ELISA reader (TECAN Sunrise, Austria). Similar to urine IgG, the OD values were transformed to antibody units by the standard curve for IgG4. Laboratory staff involved in the study had no access to the urine and serum codes or the clinical information of participants.

### Statistical analysis

The distribution of data in terms of antibody units was assessed for statistical distribution using the Kolmogorov Smirnov (K-S) test prior to performing statistical analysis. If data were not normally distributed, they were normalized by using log-transformed data, log_10_ (1 + unit). The effect of *Opisthorchis viverrini* and other parasitic infections on the antibody units for specific IgG4 and IgG in serum and urine which were not normally distributed was assessed using the Mann-Whitney *U* test. The Spearman rank correlation test was used to evaluate the correlation of specific IgG and IgG4 between urine and serum.

The cutoff values for specific IgG4 and IgG in urine and serum by *S. ratti*-based ELISA were determined by receiver-operating characteristic (ROC) analysis using Medcalc version 11.6.1.0 based on analysis of 40 proven-positive and 40 proven-negative sera samples for *S. stercoralis*. The area under the curve (AUC) indicates how well a parameter can be differentiated between two diagnostic groups, and the cutoff was calculated for highest sensitivity and specificity [12, 32, 33]. The cutoff values (antibody units/ml of urine or serum) of specific IgG4 were 12.35 in urine and 295.1 in serum. For specific IgG, the cutoffs were 184.3 and 251.4 in urine and serum, respectively. The positive rates by different indexed tests were compared using the McNemar test. Moreover, the cutoff of *S. stercoralis*-based ELISA (Ss-based ELISA) was 208.1 and NIE-based ELISA was 251.1.

Diagnostic sensitivity and specificity of specific IgG and IgG4 in serum, urine and APC and FECT were calculated using the combined results of APC and/or FECT for the fecal positive strongyloidiasis and were used as a reference standard. The sensitivity and specificity of each test were calculated using Medcalc version 11.6.1.0. The data for sensitivity and specificity were compared using the McNemar test. Pairwise comparisons of AUC values were evaluated by DeLong’s test [34].

Agreement of diagnostic methods was evaluated using Cohen's kappa coefficient (κ), for which a value < 0.2 is considered to indicate slight agreement, 0.21–0.40 is fair agreement, 0.41–0.60 is moderate agreement, 0.61–0.80 is substantial agreement, and > 0.80 is almost a perfect agreement [30, 31]. The statistical analyses on data obtained in this study were done using SPSS v.26.0. The results of statistical tests were considered significant when *P* < 0.05.

## Results

### Demographic characteristics of study participants and parasitic infections

The positive rate of *S. stercoralis* according to APC was 43.1% (75 out of 174 samples), and it was 20.4% by FECT (46 out of 226 samples). Within the APC-positive samples (75 samples), 28 samples (37.3%) were also positive by FECT while 47 samples (62.7%) were positive only by APC. Six samples (13%) positive by FECT but were negative by APC. The overall prevalence of *S. stercoralis* infection was 41.2% based on positive results by APC or FECT (93 out of 226 individuals). The intensity of infection estimated by FECT (geometric mean ± SE, *n* = 46) was 12.6 ± 3.4 larvae per gram of feces. Other parasitic infections included 61 individuals with *O. viverrini* (27.0%)*,* 8 with *Taenia* spp. (3.5%) and 2.7% with hookworms and minute intestinal flukes, 3 with *Echinostoma* spp. (1.3%) and 1 with *Trichuris trichiura* (0.4%).

For immunological analysis, the participants were divided into three groups according to fecal examination by APC and FECT (Table 1). Group 1 included *S. stercoralis*-infected individuals and individuals who had concurrent infection of *S. stercoralis* and other parasites (*n* = 93), Group 2 included people with other parasite infections who were negative for *S. stercoralis* by fecal examination (*n* = 40) including 37 individuals with *O. viverrini*, 4 with *Taenia* spp., 3 with *Echinostoma* spp. and 1 with minute intestinal fluke and hookworm; Group 3 consisted of parasite-negative individuals (*n* = 93).

**Table 1:** Demographic characteristics and parasitic infections determined by fecal examinations (FECT and APC).

Variables	*N*	%
Demographic data		
Age		
< 40	11	4.9
< 50	43	19.0
< 60	72	31.9
> 60	100	44.2
Sex		
Males	108	47.8
Females	118	52.2
Parasitic infections		
Group 1: Proven strongyloidiasis		
FECT (n = 93)	46	49.5
APCT (n = 81)	75	80.6
Total	93	41.2
Group 2: Other parasites^a^		
*Opisthorchis viverrini*	37	92.5
*Taenia* spp.	4	10.0
Hookworms	3	7.5
Minute intestinal flukes	1	2.5
*Trichuris trichiura*	1	2.5
Total	40	17.7
Group 3: Parasite negative	93	41.2
Total	226	–

^a^Some participants of this group were infected with more than one parasite species

### Detection rates of specific IgG and IgG4 in urine and serum

The numbers of individuals in each group testing positive for strongyloidiasis using total IgG and IgG4 (226 individuals tested) are shown in Additional file 1: Table S1. The detection rates in proven strongyloidiasis cases (Group 1) using IgG4 in urine (91.4%) and serum (93.5%) were similar (McNemar test, $\chi$^2^ = 0.604, df = 1, *P* > 0.05). For specific IgG in serum and urine, detection of *S. stercoralis* infection was 100% and 97.8%, respectively, and these were similar (McNemar test, $\chi$^2^ = 2.022, df = 1, *P* > 0.05). In the analysis of samples from people with other parasitic infections (Group 2), the positive rates by IgG4 in urine and serum were similar (7.5–12.5%) (McNemar test, $\chi$^2^ = 1.287, df = 1, *P* > 0.05), and those of IgG in urine and serum (10.0–12.5%) (McNemar test, $\chi$^2^ = 0.635, df = 1, *P* > 0.05), but these were not significantly different. In parasite-negative individuals (Group 3, Additional file 1: Table S1), the detection rates of IgG4 were 6.5% in urine and 7.5% in serum (McNemar test, $\chi$^2^ = 21.8, df = 1, *P* > 0.05). For IgG detection, the positive rates were 11.8% in urine and 7.5% in serum (McNemar test, $\chi$^2^ = 49.3, df = 1, *P* > 0.05).

Overall, the positive detection rates using IgG4 in urine (41.6%) and serum (43.8%) were similar (McNemar test, $\chi$^2^ = 135.7, df = 1, *P* > 0.05). Similar positive rates were also observed for IgG in urine (46.9%) and serum (46.5%) (McNemar test, $\chi$^2^ = 169.8, df = 1, *P* > 0.05). When considering the different types of immunoglobulins used to test the same clinical specimens, IgG returned higher positive rates than IgG4 in urine samples (McNemar test, $\chi$^2^ = 160.95, df = 1, *P* < 0.01).

### Diagnostic performances of IgG4 and IgG detection in urine and serum samples

The performances of indexed tests, i.e. specific IgG4 and IgG in serum and urine for diagnosis of strongyloidiasis as well as the standard parasitological methods, are shown in Table 2. With reference to positive fecal examination (individuals positive by FECT and/or APC, *n* = 93), the sensitivity of IgG4 in urine (91.4%) was similar to that of IgG4 in serum (93.6%). For estimations of diagnostic specificity based on the other parasitic infection in Group 2 (*n* = 40) and endemic negative participants in Group 3 (*n* = 93), the specificity of IgG4 in urine (93.2%) was slightly higher than that in serum (91.0%) with no significant difference (McNemar test, $\chi$^2^ = 25.5, df = 1, *P* > 0.05). For specific IgG in urine and serum, the sensitivities were similar (97.9–100%). The specificity of specific IgG in serum was 91.0% and that in urine was 88.7% (McNemar test, $\chi$^2^ = 29.2, df = 1, *P* > 0.05). The sensitivity of FECT was 49.5% and 92.6% for APC.

**Table 2:** Summary of the diagnostic performance of IgG and IgG4 ELISA in urine and serum compared with fecal examination as a reference diagnostic method

Antibody isotopes	*n*	Sensitivity (95% CI)	*n*	Specificity (95% CI)
Urine IgG4	93	91.4 (83.8–96.2)	133	93.2 (87.5–96.9)
Serum IgG4	93	93.6 (86.5–97.6)	133	91.0 (84.8–95.3)
Urine IgG	93	97.9 (92.5–99.7)	133	88.7 (82.1–93.6)
Serum IgG	93	100.0 (96.1–100.0)	133	91.0 (84.8–95.3)

The AUCs calculated by ROC analysis for IgG4 in urine (AUC = 0.923, 95% CI 0.882–0.964) and in serum (AUC = 0.923, 95% CI 0.882–0.963) were similar (DeLong’s test, *P* > 0.05). The AUCs of IgG in urine (AUC = 0.933, 95% CI 0.897–0.969) and in serum (AUC = 0.955, 95% CI 0.926–0.984) were also similar (DeLong’s test, *P* > 0.05).

The diagnostic agreement between IgG4 detection and fecal examination (combined APC and FECT) were assessed using Cohen’s kappa coefficient (*κ*) (Table 3). This revealed almost perfect agreements between fecal examination and urine IgG4 (Cohen’s kappa coefficient, *κ* = 0.845, 95% CI 0.770–0.910, *P* < 0.001) and serum IgG4 (Cohen’s kappa coefficient, *κ* = 0.837, 95% CI 0.762–0.911, *P* < 0.001). The agreement between urine IgG4 and serum IgG4 was substantial (Cohen's kappa coefficient, *κ* = 0.774, 95% CI 0.686–0.855, *P* < 0.001). For IgG in Table 4, the agreement with fecal examination (APC and FECT) was almost perfect for urine (Cohen’s kappa coefficient, *κ* = 0.848, 95% CI 0.777–0.911, *P* < 0.001) and serum IgG (Cohen’s kappa coefficient, *κ* = 0.892, 95% CI 0.832–0.946, *P* < 0.001). Urine IgG and serum IgG had almost perfect agreement (Cohen's kappa coefficient, *κ* = 0.867, 95% CI 0.796–0.929, *P* < 0.001).

**Table 3:** Diagnostic agreements between pairs of methods between fecal examination and between IgG4 in serum and urine

Variable	Kappa	95% CI		*P*-value
		Lower	Upper	
Fecal examination and urine IgG4	0.845	0.770	0.910	*P* < 0.001
Fecal examination and serum IgG4	0.837	0.762	0.911	*P* < 0.001
Urine IgG4 and serum IgG4	0.774	0.686	0.855	*P* < 0.001

**Table 4:** Diagnostic agreements between pairs of methods between fecal examination and between IgG in serum and urine

Variable	Kappa	95% CI		*P-*value
		Lower	Upper	
Fecal examination and urine IgG	0.848	0.777	0.911	*P* < 0.001
Fecal examination and serum IgG	0.892	0.832	0.946	*P* < 0.001
Urine IgG and serum IgG	0.867	0.796	0.929	*P* < 0.001

### Cross-reactions in samples from individuals with *O. viverrini* and other parasitic infections

**Fig. 2:**
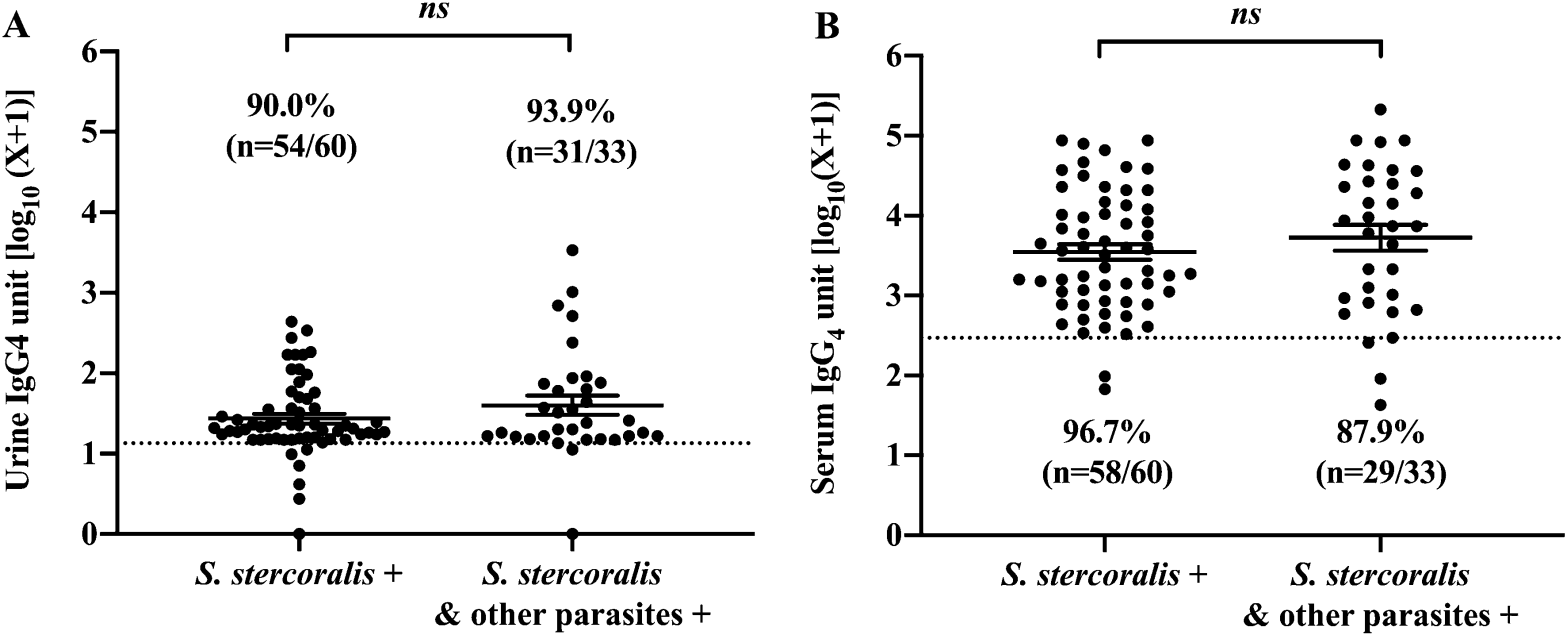
Measurements of IgG4 in urine (**A**) and IgG4 in serum (**B**) in individuals with parasitologically confirmed strongyloidiasis only (*n* = 60) and those with mixed infections of *Strongyloides stercoralis stercoralis* and other parasitic infections (*n* = 33). There was no significant difference in antibody levels between these groups for IgG4 in both serum and urine. The dotted line indicates the cutoff value of each diagnostic method

Since *O. viverrini* is also endemic in the study area, it is necessary to examine whether infection by *O. viverrini* as well as other parasitic infections can influence results of serum and urine ELISA. Antibody levels (*Strongyloides*-specific IgG4 and IgG) were compared between samples positive for *S. stercoralis* alone and samples positive for *S. stercoralis* with other concurrent parasite infections. Among the strongyloidiasis participants (Group 1, *n* = 93), 33 individuals were co-infected with *O. viverrini* and other parasites. There were no differences in levels of IgG4 antibody in urine between the mono-infection of *S. stercoralis* and those with mixed infection of *S. stercoralis* with other parasites (Mann-Whitney *U*-test, *U* = 891, Z = − 0.795, *P* = 0.4296). (Fig. 2A). Similar results were seen for IgG4 in serum between *S. stercoralis* infection alone and those with *S. stercoralis* mixed with other parasites infection (Mann-Whitney *U*-test, *U* = 854.5, *Z* = − 1.088,* P* = 0.2790) (Fig. 2B).

**Fig. 3:**
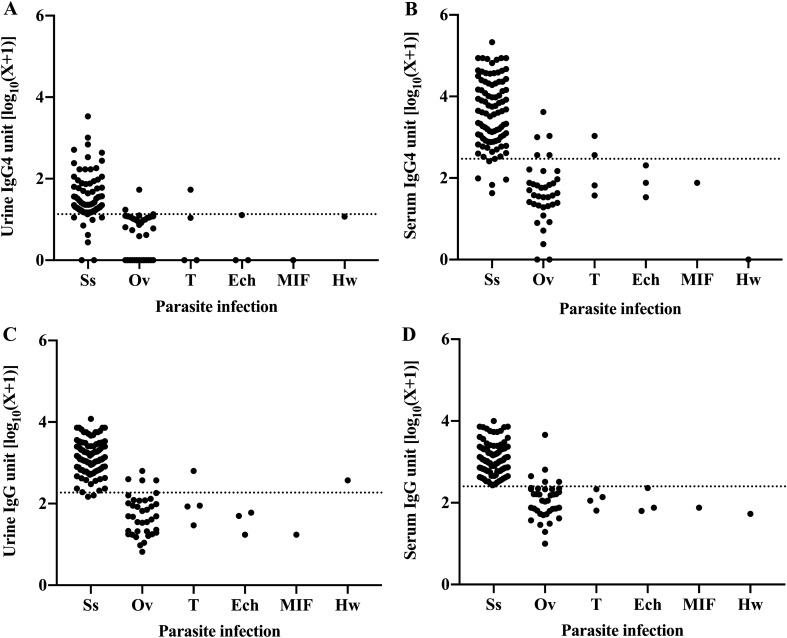
Levels of *Strongyloides*-specific antibodies in samples from individuals infected with *Strongyloides stercoralis* and other parasites. The levels of urine IgG4 (**A**), serum IgG4 (**B**), urine IgG (**C**) and serum IgG (**D**) in individuals infected with Ss *S. stercoralis*, Ov *Opisthorchis viverrini*, Ech *Echinostoma* spp., T *Taenia* spp., MIF minute intestinal flukes, Hw hookworm. The dotted line indicates the cutoff value of each diagnostic method

To observe the cross-reactivity of IgG and IgG4 detection in serum and urine to other parasitic infections (Group 2; *n* = 40), participants parasitologically confirmed to have other parasitic infections were analyzed. The parasites detected in Group 2 were *O. viverrini* (*n* = 37), *Taenia* spp. (*n* = 4), *Echinostoma* spp. (*n* = 3), minute intestinal flukes (*n* = 1) and hookworm (*n* = 1). Cross-reactivity of specific IgG4 was observed in 3 of 37 individuals (8.1%) in urine and 5 of 37 individuals (13.5%) in serum with *O. viverrini*. Cross-reactivity in *Taenia* spp. infection occurred in one of four participants (25.0%) in urine IgG4 and two of four participants (50.0%) in serum IgG4 (Fig. 3A, B). In cases of IgG detection, cross-reactions were found in 4 of 37 individuals (10.8%) in urine and 5 of 37 individuals (13.5%) in serum with *O. viverrini*. Detection of IgG was found to be positive in one of five individuals with *Taenia* spp. in urine (25.0%). One participant with hookworm infection was found to be positive with IgG in urine but not in serum (Fig. 3C, D). No cross-reaction with a case of hookworm infection was found for IgG4 in urine or serum.

### Correlation between IgG4 and IgG antibody units in urine and serum

**Fig. 4:**
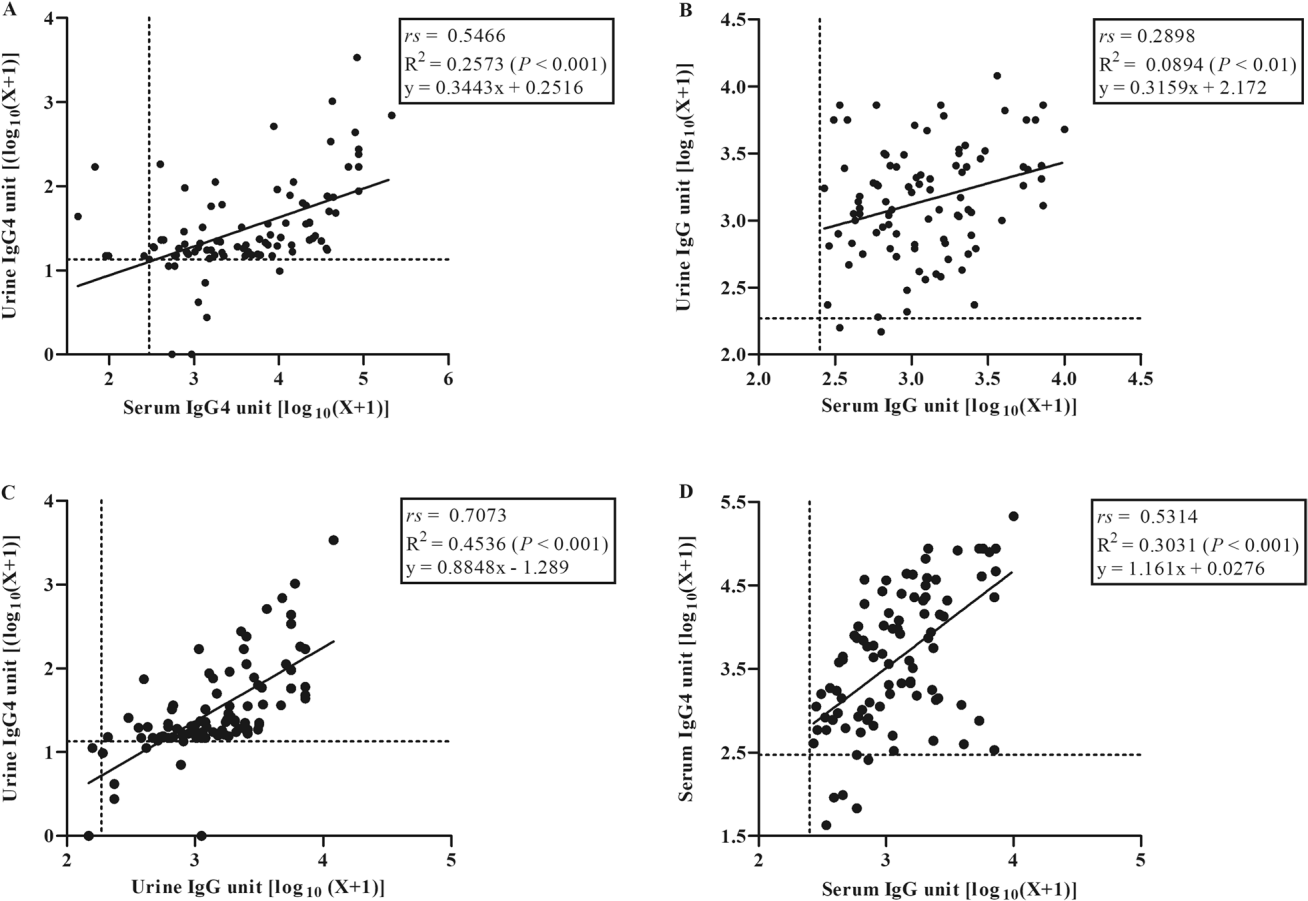
Correlations between levels of IgG4 in urine and serum (**A**), IgG in urine and serum (**B**), between IgG and IgG4 in urine (**C**) and between IgG and IgG4 in serum (**D**). Coefficients of correlation were determined using the Spearman correlation test (*rs*). Data shown are observed values, and the solid line was calculated from the regression equation (Y = ax + b, Y = log IgG/IgG4, a = slope, x = log IgG/IgG4, b = Y-intercept). Dotted lines (vertical and horizontal) represent the cutoff values

The relationships between specific IgG4 and IgG antibody in serum and urine among fecal positive strongyloidiasis were analyzed (*n* = 93) (Fig. 4). A statistically significant correlation was found between the level of IgG4 in urine and serum (Spearman’s correlation coefficient, *rs* = 0.5466, *P* < 0.001) (Fig. 4A). There was a similar correlation between the levels of IgG in urine and serum (Fig. 4B) (Spearman’s correlation coefficient, *rs* = 0.2898, *P* < 0.01). In addition, there were significant positive correlations between IgG and IgG4 in urine (Spearman’s correlation coefficient, *rs* = 0.7073; *P* < 0.001, Fig. 4C) and in serum (Spearman’s correlation coefficient, *rs* = 0.5314; *P* < 0.001, Fig. 4D).

## Discussion

Sensitive and specific methods for diagnosis of strongyloidiasis are required to compensate for the low diagnostic sensitivity of fecal examination. As a follow-up to a previous study on detection of *Strongyloides*-specific IgG in urine [11], we analyzed IgG4 in urine for diagnosis of strongyloidiasis. Levels of *Strongyloides*-specific IgG in urine and serum were also determined to evaluate the diagnostic performance of these immunological methods and for comparison against the standard parasitological methods (FECT and APC). The results showed that detection of IgG4 in urine had a similar diagnostic sensitivity to that of IgG4 in serum (93.6% vs. 91.4%) and had similar diagnostic specificity (93.2% vs. 91.0%). Detection of specific IgG4 in urine and serum had strong agreement with results from fecal examination (Cohen’s kappa coefficient was approximately 0.8). There were significant positive correlations between IgG4 levels (as well as total IgG) in urine and in serum. IgG4 in urine and serum had lower cross-reactivity with other parasitic infections than did IgG. The results suggested that urine IgG4 has potential to serve as another IgG immunoglobulin subclass for immunological diagnosis of strongyloidiasis. In addition, from the interview of the participants, there was no history of deworming activity by anthelminthic drug in the participants in the study community. Hence, there should be no interference of drug treatment in the comparison between parasitology and serology in this study. Indeed, it is the first time that the participants who were positive for strongyloidiasis by parasitological and serological method received ivermectin treatment.

Regarding the dynamics of these antibodies in strongyloidiasis, there were reports that the level of specific IgG in serum significantly decreased at 1 year post treatment in *S. stercoralis*-infected individuals cured from drug treatment (based on negative fecal tests and < 3 positive results out of the 5 serologic tests for serodiagnosis) [32]. Similarly, the antibody responses (IgG and IgG4) in strongyloidiasis individuals measured by Ss-NIE/Ss-IR-based ELISA exhibited a statistically significant decline in most of the patients over 1 year post chemotherapy, and a few patients converted to negative serology [33]. Whether there is a similar trend of decline in specific-IgG in urine at post drug treatment and seroconversion or not requires further investigation.

Since the participants in group 2 and 3 came from the same endemic area as those *Strongyloides* positive in group 1, it is likely that they included a considerable proportion of false-negative diagnoses by a single fecal examination which was used in this study. Thus, the seropositive tests found in individuals in group 2 and 3 were anticipated. Moreover, caution in interpretation of data in intensity of infection for strongyloidiasis is needed because of a low sensitivity of FECT in detecting *S. stercoralis* larva in feces. Hence, the estimated intensity of infection (larvae/gram feces) is restricted to larval positive individuals and biased toward individuals with high larval counts.

Nematode infections, including strongyloidiasis and filariasis, have been reported to stimulate a high IgG4 response associated with asymptomatic and chronic infections [17–20]. Repeated or long-term exposure to the same antigen of filariasis and strongyloidiasis induced IgG subclass switching to specific IgG4 [34, 35]. IgG4 may aid parasite evasion of the host immune response by induction of immune tolerance [36]. This may be done via the immune modulator IL-10 and TGF-β-secreted Tregs, which alter the activity of antigen-presenting cells (APCs) or interfere with antigen presentation between the APC and the T cell [37]. Moreover, a predominance of IgG4 can counteract IgE responses [38, 39] leading to reduced activation of antibody‐dependent cell‐mediated cytotoxicity (ADCC) [40]. IgG4 is also known to compete with IgE for the antibody-fixation sites on mast cells and eosinophils leading to inhibition of degranulation [39, 41], Thus, IgG4 plays important roles in chronic strongyloidiasis and have received considerable attention in serodiagnosis.

Use of *S. ratti* as heterologous antigen for serodiagnosis of strongyloidiasis is preferable compared to *S. stercoralis* because of the ease and safety of antigen preparation and also high sensitivity [12]. Moreover, in case of the recombinant antigen of *S. stercoralis* (i.e.NIE), it is not widely available for mass screening in the endemic field setting. In support of using *S. ratti* antigen, our results showed that concurrent infection of *S. stercoralis* with *O. viverrini* has no effect on serodiagnosis of strongyloidiasis by IgG4 from *S. ratti*-based ELISA. The result that IgG in urine had higher sensitivity than IgG4 in urine may relate to the higher concentration of IgG than IgG4 antibody in urine and also in serum. The utility of *Strongyloides*-specific IgG4 as a serum biomarker for diagnosis of human strongyloidiasis has been widely reported using different platforms including the lateral-flow assay method [22–25] and ELISA [20, 33]. It had been reported that the sensitivities of IgG4 were lower than those of IgG but the specificity was higher (i.e. increased specificity by 13.3%) [18]. Detection of *Strongyloides*-specfic IgG4 by ELISA based on *S. stercoralis* antigens showed sensitivity of 96% and specificity of 93%; therefore, this method is suitable for immunodiagnosis of strongyloidiasis in alcoholic and non-alcoholic individuals [42]. Using recombinant antigens (NIE/SsIR), the IgG- and IgG4-based assays yielded sensitivities of 92% and 81% and specificities of 91% and 94%, respectively [20]. Mixed recombinant antigen ELISAs incorporating Ss-NIE/Ss-IR for IgG- and IgG4 detections yielded similar sensitivity and specificity [33]. The result that IgG in urine had higher sensitivity than IgG4 in urine (shown in Additional file 1: Table S1) may be due to the higher serum concentrations of IgG than IgG4 [35], and it is expected that a similar situation occurred in IgG4 in urine. Nevertheless, the results in our study suggest that urine IgG4 can be quantitatively measured and provides similar diagnostic performance to serum IgG4 for diagnosis of strongyloidiasis.

We detected cross-reactions for *Strongyloides*-specific IgG4 in 3 of 37 individuals (8.1%) in urine and 5 of 37 individuals (13.5%) in serum from persons with *O. viverrini*. Similar rates of cross-reaction with *Taenia* spp. were observed in urine (1 of 4) and in serum (2 of 4). For *Strongyloides*-specific IgG, cross-reaction with *O. viverrini* was observed in 4 of 37 (10.8%) urine samples and 5 of 37 (13.5%) serum samples. Similar cross-reactivity of serum IgG with samples from *O. viverrini* infection (13–25%) were reported [11, 12]. In the case of IgG4, a previous study also reported that IgG4 in serum used to diagnose strongyloidiasis cross-reacted with filariasis (4/55 participants): cross-reactivity was higher (10/55) for IgG (9 filariasis and 1 trichostrongyliasis patients) [18]. These results were interpreted as possibly indicating concurrent strongyloidiasis with other parasitic infections, particularly *O. viverrini*, which is frequently found in Northeast Thailand [28]. Another possible reason is that *S. stercoralis* infection may be present but was not detected by fecal examination. Indeed, the cross-reactive cases with *O. viverrini* for IgG4 in urine and serum yielded low-positive results since the antibody levels were < 10% above the cutoff values. There were lower cross-reaction rates for IgG4 in urine and serum than for IgG in the same clinical specimens. Clearly, the paucity of additional cross-reactivity studies on other parasite species (e.g. *Schistosoma mansoni*, hookworms, filarial nematodes, *T. trichura*) is a limitation of our study, and more intensive cross-reactivity should be further investigated.

The pathway by which *Strongyloides*-specific antibodies reaches urine is not fully known. Theoretically, these antibodies could be derived from the immune complex localized in kidney and induce pathology in the glomerulus by complement activation, leukocyte chemoattraction and inflammation, leading to damage of the glomerular barrier and allowing macromolecules to pass into urine [43–45]. Whatever the case, antibody detection in urine in strongyloidiasis cases is consistent and reliable for diagnosis, with diagnostic values comparable to those based on serum detection [11, 12] and in opisthorchiasis [43].

## Conclusion

Human strongyloidiasis is a serious public health problem that requires early detection with highly efficient but simple diagnostic methods. Our findings suggest that detection of IgG4 in urine using an ELISA platform is an alternative diagnostic tool for human strongyloidiasis that shows similar performance in terms of sensitivity and specificity to detection of serum IgG4. The collection of urine samples is noninvasive, and urine samples are much easier to handle than blood samples. Urine ELISA can be used for routine laboratory analyses, epidemiological studies, and implementation of public health control programs against strongyloidiasis. Further studies are required to evaluate the accuracy of IgG4 biomarkers from urine for measuring the efficacy of anthelmintic treatment with ivermectin on *S. stercoralis* infection in endemic rural communities.

### Supplementary Information

**Additional file 1: Table S1** Positive rates of *Strongyloides stercoralis* determined by specific IgG4 and IgG detection in different parasitic infection groups based on fecal examination (APCT and FECT).

## Data Availability

The datasets generated and/or analyzed during the current study are available from the corresponding author on reasonable request.

## Abbreviations

ELISA: Enzyme-linked immunosorbent assay
APC: Agar plate culture technique
FECT: Formalin-ethyl acetate concentration technique
IgG: Immunoglobulin G
IgG4: Immunoglobulin G4
κ: Cohen's kappa coefficient
*r*: Correlation coefficient
95% CI: 95% Confidence intervals
AUC: Area under the curve
ROC: Receiver operating characteristic
