# Supplementary material for: Diagnostic performance of Strongyloides-specific IgG4 detection in urine for diagnosis of human strongyloidiasis
Source: Parasit Vectors. 2023 Aug 28;16:298. doi: 10.1186/s13071-023-05935-6 (PMC10464225; doi:10.1186/s13071-023-05935-6)
Supplement: Supplementary file 1 — Additional file 1: Table S1 Positive rates of Strongyloides stercoralis determined by specific IgG4 and IgG detection in different parasitic infection groups based on fecal examination (APCT and FECT). [file 13071_2023_5935_MOESM1_ESM.docx]

**Table S1** Positive rates of *S. stercoralis* determined by specific IgG4 and IgG detection in

different parasitic infection groups based on fecal examination (APCT and FECT).

| **Status of**  **parasitic infection** | **N** | **Numbers in each group testing positive for strongyloidiasis using different diagnostic methods (%)** | | | | |
| --- | --- | --- | --- | --- | --- | --- |
|  |  | **IgG4 detection** | | **IgG detection** | | |
|  |  | **Urine** | **Serum** | **Urine** | **Serum** | |
| **Group 1**  Proven strongyloidiasis | 93 | 85 (91.4) | 87 (93.5) | 91 (97.8)^*^ | 93 (100.0) |  |
| **Group 2**  Other parasites | 40 | 3 (7.5) | 5 (12.5) | 4 (10.0) | 5 (12.5) |  |
| **Group 3**  Parasite negative | 93 | 6 (6.5) | 7 (7.5) | 11 (11.8) | 7 (7.5) |  |

* Significance difference between urine IgG and IgG4 according to the McNemar test ($\chi$^2^=21.7, df=1, *P* < 0.05)
